# Impact of implementing a competency-based job framework for clinical research professionals on employee turnover

**DOI:** 10.1017/cts.2020.22

**Published:** 2020-03-11

**Authors:** Marissa Stroo, Kirubel Asfaw, Christine Deeter, Stephanie A. Freel, Rebecca J. N. Brouwer, Betsy Hames, Denise C. Snyder

**Affiliations:** Duke University School of Medicine, Durham, NC 27705, USA

**Keywords:** Clinical research, workforce, employee turnover, competency-based jobs, retention

## Abstract

**Introduction:**

A new competency-based job framework was implemented for clinical research professionals at a large, clinical research-intensive academic medical center. This study evaluates the rates of turnover before and after implementation of the new framework. Turnover in this workforce (as with most) is costly; it contributes to wasted dollars and lost productivity since these are highly specialized positions requiring extensive training, regardless of experience in the field.

**Methods:**

Trends in employee turnover for 3 years prior to and after the implementation of competency-based job framework for clinical research positions were studied using human resources data. Employee demographics, turnover rates, and comparisons to national statistics are summarized.

**Results:**

Employee turnover within the clinical research professional jobs has decreased from 23% to 16%, a 45% reduction, since the implementation of competency-based job framework.

**Conclusion:**

The new jobs and career ladders, both of which are centered on a competency-based framework, have decreased the overall turnover rate in this employee population. Since little is known about the rates of turnover in clinical research, especially in the academic medical setting, the results of this analysis can provide important insights to other academic medical centers on both employee turnover rate in general and the potential impact of implementing large-scale competency-based job changes.

## Introduction

Clinical research site quality is an important contributor to the clinical research enterprise. One component of site quality is the retention of employees working at the sites [1–4]. Retaining skilled clinical research professionals (CRPs) is a critical factor in improving the overall quality of research. A 2017 study of CRPs in the area of pediatric oncology found that 37% of respondents were categorized as “at risk” of leaving their position within 6 months [5]. Despite the importance of employee retention, turnover in clinical research, especially in academic settings, has not been well studied. Currently, there are no available data for turnover rates and trends for CRPs among those employed in academic medical centers (AMCs). The closest comparisons with available data are healthcare and contract research organizations (CROs). The turnover in healthcare is highly variable with annual rates for total turnover since 2013 ranging from 16.8% [6] to 33.0% [7], which may depend on who is reporting the data, how turnover is defined, and what is included as part of the healthcare sector. The Bureau of Labor Statistics put the healthcare turnover rate for 2018 at 33.0% up from 30.2% in 2014 [7]. Healthcare may be too large and too vaguely defined to use as a comparison for clinical research. CROs report turnover rates of 20% or higher in 7 of the past 10 years [8]; however, it is difficult to compare how staffing trends at CROs may or may not be relevant to AMC environments.

**Fig. 1.**
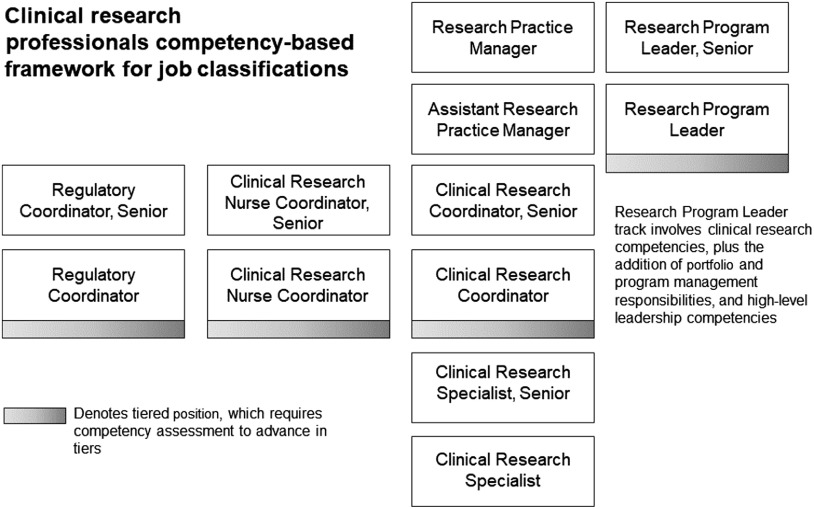
Current competency-based clinical research professional positions.

In an effort to professionalize clinical research support, the Joint Task Force (JTF) for Clinical Trials Competency spearheaded competency development in 2013 [9]. The JTF set forth core competencies that cover the types of tasks CRPs may do as a part of their daily work. Duke University Schools of Medicine and Nursing began adapting and implementing the core competencies for their work environment. That work led to the creation of 12 new competency-based job descriptions for CRPs at Duke. Fig. 1 presents the 12 CRP positions. In September 2016, all identified clinical research staff were mapped into these new jobs, reducing the job classifications from more than 80 to 12. A second round of mapping was completed in March 2017 to map employees who were missed during the first round or who may have been hired in the interim. Details of these methods were published previously [10].

Clinical research is a highly regulated field. This requires CRPs to undergo extensive training and necessitates a great deal of oversight, especially for employees who are new to the field or site. Research studies often have aggressive timelines for enrollment and completion; this results in an environment where turnover can be especially costly, both in terms of actual dollars and lost productivity. Duke estimates this turnover cost to be roughly $25,000 per employee. The cost calculation is based on a $55,000 starting salary of a new clinical research coordinator (CRC) with benefits, a 3-month onboarding/training period where the CRC has little ability to contribute to revenue generation (13K, approximately 25% annual salary); 2K human resources (HR) costs associated with search and hire (personnel time, drug testing, background, and credentialing), and 10K senior staff time (management, training, and oversight). Turnover is not only costly, it is also associated with organizational ineffectiveness, which is the inability for an organization to achieve its goals [5].

One challenge to studying turnover in clinical research in academic medical environments may be the historic lack of clarity in who supports clinical research. Employees may have held hybrid roles or had job titles which make it difficult to determine if they were working in clinical research, such as lab manager, biostatistician, or staff specialist. The implementation of a competency-based job framework, and jobs based on that framework, may make it easier to identify the CRPs. In such a framework, the CRPs are in easily identifiable classifications, which allow straightforward tracking and reporting of trends within that population. At Duke, the process of mapping CRPs into 12 defined jobs allowed for an accurate analysis of rates of turnover (defined here as employees leaving Duke). This was an impossible task prior to having 12 discrete job classifications.

The aim of this paper is to understand trends and rates of turnover for CRP employees for the 3-year period both pre-mapping and post-mapping and to describe CRP staff employment during these periods. Additionally, this paper explores the turnover since mapping, by position, to understand what job types may have the highest rates of turnover.

## Materials and Methods

Working with Human Resources and as part of our Workforce Engagement and Resilience (WE-R) initiative, we created a representative sample dataset. Prior to mapping, CRPs were classified in more than 80 positions, most of which were not unique to clinical research staff (for additional details on the mapping process, see Brouwer *et al.* [11]), which made identifying CRPs pre-mapping very difficult. To define the pre-mapping cohort, the WE-R team identified the 14 jobs that were known, historically, to be held almost exclusively by staff associated with clinical research within the 108 organizational units that represent the Schools of Medicine and Nursing. While these positions are only a fraction of the 80+ jobs staff in clinical research held and were mapped from, the majority of those positions were only occupied by a few staff associated with clinical research and also held by many nonclinical research staff within the same organizational units, making it difficult to include in analyses. Examples of such jobs would be Staff Assistant or Lab Manager. The employee sample in this population included 80% of the staff who were initially mapped into one of the 12 new jobs and who represented the initial post-mapping sample. The WE-R team included staff and leadership who each had 10+ years of experience in clinical research at Duke. Using those 14 job classifications and 108 organizational units, a HR transaction file was derived reflecting all movement into, out of, and within the identified positions and units. Using that file, our team evaluated the attrition for three fiscal years prior to mapping, from July 1, 2013 to June 30, 2016.

Similarly, for post-mapping, a HR transaction file reflecting the 12 new CRP job classifications and the 108 units was generated monthly to aid in tracking the impact of the mapping process. Those files were combined to create a master file that reflected all employee movement since mapping was competed, and employee attrition between September 1, 2016 and August 31, 2019. This resulted in three full years of data on each end of the mapping process. The 2-month gap between the end of the pre-mapping period and the start of the post-mapping allowed for a clean analysis with no interference in the data from the mapping process.

For this study, attrition in both the pre- and post-mapping populations was defined as an employee voluntarily or nonvoluntarily leaving Duke. Voluntary turnover reasons included resignation, retirement (*n* = 44, 6.8% of total voluntary turnover), and death (*n* = 6, 0.9% of total voluntary turnover), while involuntary turnover included end of contract or funding, poor performance, and serious violations of company policies. The definitions for voluntary and nonvoluntary used in this study are based on the definitions supplied by our human resources department and are consistent with the definitions from other institutions.

In order to determine an attrition rate, new hires had to be identified – they were defined as anyone initially entering into one of the targeted job classifications after September 1, 2016. Employees moving between the targeted job classifications were excluded from the count of new hires. A total population size was calculated for both the start and the end of each fiscal year period, which reflected all movement into, or out of, the targeted jobs, including those deemed to be internal turbulence (movement of employees within the organization). Attrition rates for each fiscal year were calculated independently, using the common formula for
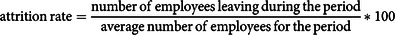



[12]

Any employees leaving Duke were flagged within the dataset, and based on their birthdate, continuous service date, position effective date, and termination date, we calculated their age, number of years at Duke, and number of years in their current position. The HR data file also contained information on gender, race, and ethnicity that were used to calculate population demographics.

In previous published work [10], we cited the initial number of mapped employees as 557. Following that initial mapping in September, an additional 141 existing employees were mapped into the CRP jobs.

## Results

The starting population in the pre-mapping sample was 445 active employees and the final pre-mapping population size was 560, while 698 employees were included in the sample at the start of the post-mapping period and there were 829 active employees at the end of the post-mapping period. The total number of employees active at any time during the pre-mapping sample was 1290, and in the post-mapping sample was 1360. Table 1 shows the demographics of the pre- and post-mapping samples, which were very similar. The average age in the samples is virtually identical. There were slight differences in the overall distribution of race and ethnicity in the samples, representing an increase in diversity. The percentage of White employees in the pool decreased from 71.4% pre-mapping to 69.4% post-mapping. There was an increase from pre- to post-mapping of 1.8% of those who identified as Hispanic (from 0.5% in the pre-mapping to 0.6% in the post-mapping sample). The sample also became slightly less female in the post-mapping period, with a decrease from 85.7% to 83.7%.

**Table 1.** Demographics for pre- and post-mapping samples

	Pre-mapping (*n* = 1290)		Post-mapping (*n* = 1360)	
	Mean	SD	Mean	SD
Age	39.3	12.7	39.7	12.8
	*N*	%	*N*	%
Gender – female	1106	85.7	1138	83.7
Race				
White	921	71.4	944	69.4
Black	219	17.0	232	17.1
Other	150	11.6	184	13.5
Ethnicity – non-Hispanic	1229	95.3	1273	93.6

SD, standard deviation.

Table 2 presents the turnover rates by year, and the 3-year annualized average, for the pre- and post-mapping periods. The turnover rates for the pre-mapping period range from 21.3% to 25.8%, with an average of 23.3%. Post-mapping, the annual turnover rates range from 14.1% to 18.1%, with an average of 16.0%. This includes all types of turnover. When restricting the analysis to voluntary turnover, the average rates for the 3-year period are 20.3% in the pre-mapping sample and 14.8% in the post-mapping sample, which is a reduction of 36.9% in voluntary turnover among the CRP population. When excluding retirement or death from the voluntary turnover, the 3-year average rates were 18.4% in the pre-mapping sample and 13.7% in the post-mapping sample, which is a reduction of 36.9%, the same as when retirement and death are included with resignations. Nonvoluntary turnover did not change from the pre- to post-mapping sample.

**Table 2.** Turnover rates pre- and post-mapping

	All turnover (%)	Voluntary turnover only (all reasons) (%)	Resignations only (excludes retirement and death) (%)
Pre-mapping			
Year 1	21.3	19.5	18.8
Year 2	25.8	21.2	19.9
Year 3	22.4	20.2	17.5
3-year annualized average	23.3	20.3	18.7
Post-mapping			
Year 1	14.1	13.7	12.3
Year 2	15.9	14.8	13.9
Year 3	18.1	16.0	14.8
3-year annualized average	16.0	14.8	13.7

**Fig. 2.**
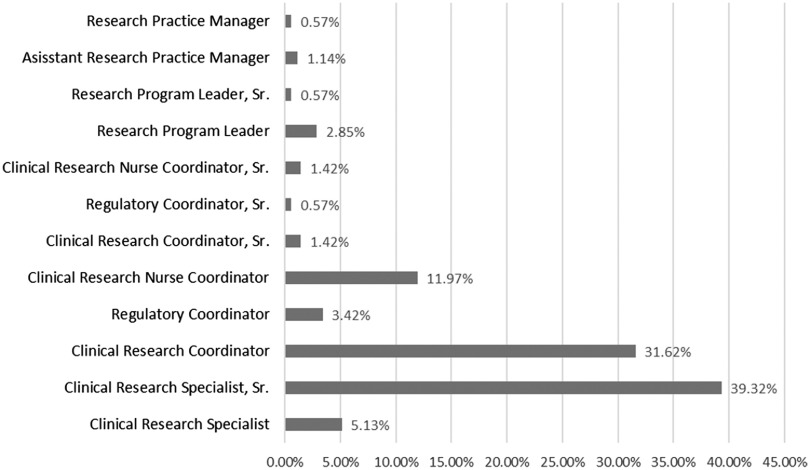
Percentage of total post-mapping turnover by position.

Fig. 2 presents the percentage of turnover, by position, for employees since the mapping process. Highest turnover is seen in the entry-level Clinical Research Specialist, Sr. and Clinical Research Coordinator positions, at 39.3% and 31.6%, respectively. The lowest turnover (0.6%) is among the higher level and leadership positions of Research Practice Manager, Research Program Leader, Sr., and Regulatory Coordinator, Sr. These data make sense, as the Clinical Research Specialist, Sr. and Clinical Research Coordinator roles are more likely to be filled by younger employees, more recently graduated, and especially in the Clinical Research Specialist, Sr. position, more likely to be looking for a gap-year job before applying to medical or graduate school. The Research Practice Manager, Research Program Leader, Sr., and Regulatory Coordinator, Sr. positions require more years of experience to apply and are often filled by employees who have made a career in clinical research. These positions also represent a smaller total number of jobs than in the Clinical Research Coordinator or Clinical Research Specialist, Sr. pool.

## Discussion

Overall, employee turnover within the CRP jobs at this AMC has decreased from 23% to 16%, a 30% reduction, since the implementation of a competency-based workforce initiative. This is a strong, positive result, and suggests that the investment in implementing competency-based jobs with clearer opportunity for career progression for CRPs may pay off by improving retention (at least in the short term). While there are short-term costs to implementing such large-scale changes, this study suggests that institutions may be actualizing an overall savings by reducing annual turnover costs.

There have been annual increases in turnover rates for this population, especially during the most recent year. However, when comparing our data to national trends in turnover and job separations, these increases are well below the overall rates but follow the trend of year-over-year increases. According to the Work Report on Employee Retention [13], 27% of employees voluntarily left their job in 2018, a number which is only expected to rise over the next few years, to a forecasted 35% in 2023.

Most promising to this organization is that the national trend has been steadily increasing since 2014, while we have experienced a dramatic decrease in 2016 following the mapping process and have not reached the previous annual turnover rates in any time period since. Observing a decrease during a time when turnover has been on the rise nationally is an encouraging sign. At our institution, the decrease in turnover was among *voluntary* turnover, which is where you would hope to see impacts from a complete overhaul of job classifications.

### Strengths and Limitations

One of the strengths of this study was the large sample size from human resource data for a large AMC. While the sample represents only one workplace, it represents a large clinical research workforce in a very competitive area for clinical research. Anecdotally, based on knowledge of the local climate and professional contacts in the area, turnover in this area of North Carolina may be higher than in other areas of the country, due to the large number of positions in clinical research in both the academic, CROs, and industry organizations. The 2018 North Carolina Annual Economic did note the increasing opportunities within the state for job seekers, with only 1.9 jobseekers per opening in 2018 [14]. The unemployment data for the region of the state that includes Duke are even lower, at 4.4% in 2017, compared to 4.6% for the entire state [15]. Relying on human resource data may also be a limitation, especially in the pre-mapping sample, where identifying employees was difficult. Job titles for staff were not always unique to clinical research, and many CRPs were not working in positions that suggested that their primary activities were related to clinical research. This led to limiting our pre-mapping sample to only those positions most clearly held by CRPs that matched to about half of post-mapping sample size. However, we believe that the pre-mapping turnover rates closely reflect reality.

We are also limited by the lack of additional contextual data about turnover reasons. We can only classify the employee’s reason for leaving as voluntary or nonvoluntary. Voluntary turnover may be due to reasons that cannot be impacted by the new job classifications and career ladders, such as returning to school to pursue advanced degrees or moving for a spouse or other noncareer-related reasons for the employee. An optional exit survey process has been implemented and should provide an opportunity for further exploration of employee turnover among our CRPs.

While there are no available published turnover rates for CRPs at AMCs to which we can compare our data, we also acknowledge that our rate may be higher than other sites, as Duke is located in a very competitive location for clinical research. There are two other AMCs within close proximity, as well a number of CROs, nonacademic research companies, and pharmaceutical companies that may compete for the same employees. Real or perceived availability of other jobs and opportunities for career advancement is one external factor that influences turnover [5, 16]. A study on turnover and local job markets found that employment opportunities were a relevant factor in areas with a higher population [17].

### Future Directions

While the decrease in turnover has been substantial, efforts are underway to further increase retention of this valuable workforce. One substantial effort is a redesign of our employee onboarding process. Onboarding is defined as “the process of helping new hires adjust to social and performance aspects of their jobs” [18]. The social aspects are as important as the performance aspects. Bauer identified four levels of onboarding, from compliance at the bottom to connection at the top. Connection is defined as building formal and informal relationships, and employees who reported having an onboarding process that met the criteria to be considered effectively onboarded to the connection level reported more job satisfaction [19], and job satisfaction is a key reason why employees remain in a job [20].

Another benefit of a redesigned onboarding process will be the opportunity to educate all new CRPs about opportunities for career advancement within clinical research at this institution. In the study of research staff within the areas of pediatric oncology, one of the primary motivators to look for other work was a lack of opportunities for career advancement and professional development [5]. Having a clearly defined career ladder and opportunities for professional development may increase the perceived potential forfeited costs of leaving a company [21] and that may increase retention. We have continued to support professional development of our workforce through the Research Professionals Network at Duke. A Clinical and Translational Science Award-funded initiative aims to 1) provide professional development through workshops, lectures, and discussion groups; 2) foster networking internally and externally to Duke clinical research; and 3) champion a culture of excellence [22]. Also, our WE-R team will continue to advocate to support professional development opportunities (certification and conference participation) which further supports employee career development. Further analysis of exit survey data on the reasons why employees leave may also help identify new areas to explore to reduce turnover.

One future area of study is to evaluate institutional turbulence (defined as movement of employees within Duke). This paper evaluates only the rates of employees leaving the institution, which is just a part of the story. Internal employee movement can also negatively affect costs and, more importantly, overall study quality. Abshire *et al.* [23] noted that participant retention is impacted by having high-quality staff who are specialized and demonstrate good team work. These qualities are likely the result of employees who have remained within the same research unit for a period of time.

Ultimately, our vision is to evaluate the longer term turnover and retention data and determine the impacts of this on measures of site quality, such as study enrollment, participant retention, data quality, and protocol deviations.

## Conclusion

While much work remains to understand the full impact of the competency-based jobs framework implemented at Duke, early signs are positive. A 30% reduction in turnover, during a time period where national trends were consistently increasing, is meaningful. It is too early to evaluate how this may relate to site quality, but we believe that retaining trained and competent site staff for clinical research will result in lower costs, quality clinical research, and fulfilling careers. The work undertaken by Duke has been widely shared and is being adapted and adopted by other AMC research sites. In time, we hope other institutions will publish data on their CRP turnover rates to help advance the field and learn what may improve retention and increase the quality of clinical research.
